# Relapse in resected lung cancer revisited: does intensified follow up really matter? A prospective study

**DOI:** 10.1186/1477-7819-7-87

**Published:** 2009-11-12

**Authors:** Dragan Subotic, Dragan Mandaric, Gordana Radosavljevic, Jelena Stojsic, Milan Gajic, Maja Ercegovac

**Affiliations:** 1 Institute for Lung Diseases, Clinical Center of Serbia, Belgrade, Serbia; 2 Institute for Medical Statistics, Faculty of Medicine, Belgrade, Serbia

## Abstract

**Background:**

beside the well known predominance of distant vs. loco-regional relapse, several aspects of the relapse pattern still have not been fully elucidated.

**Methods:**

prospective, controlled study on 88 patients operated for non-small cell lung cancer (NSCLC) in a 15 months period. Stage IIIA existed in 35(39.8%) patients, whilst stages IB, IIA and IIB existed in 10.2%, 4.5% and 45.5% patients respectively. Inclusion criteria: stage I-IIIA, complete resection, systematic lymphadenectomy with at least 6 lymph node groups examined, no neoadjuvant therapy, exact data of all aspects of relapse, exact data about the outcome of the treatment.

**Results:**

postoperative lung cancer relapse occurred in 50(56.8%) patients. Locoregional, distant and both types of relapse occurred in 26%, 70% and 4% patients respectively. Postoperative cancer relapse occurred in 27/35(77.1%) pts. in the stage IIIA and in 21/40(52.55) pts in the stage IIB. In none of four pts. in the stage IIA cancer relapse occurred, unlike 22.22% pts. with relapse in the stage IB. The mean disease free interval in the analysed group was 34.38 ± 3.26 months.

The mean local relapse free and distant relapse free intervals were 55 ± 3.32 and 41.62 ± 3.47 months respectively Among 30 pts. with the relapse onset inside the first 12 month after the lung resection, in 20(66.6%) pts. either T3 tumours or N2 lesions existed. In patients with N0, N1 and N2 lesions, cancer relapse occurred in 30%, 55.6% and 70.8% patients respectively

Radiographic aspect T stage, N stage and extent of resection were found as significant in terms of survival. Related to the relapse occurrence, although radiographic aspect and extent of resection followed the same trend as in the survival analysis, only T stage and N stage were found as significant in the same sense as for survival. On multivariate, only T and N stage were found as significant in terms of survival.

Specific oncological treatment of relapse was possible in 27/50(54%) patients.

**Conclusion:**

the intensified follow up did not increase either the proportion of patients detected with asymptomatic relapse or the number of patients with specific oncological treatment of relapse.

## Background

Despite the well known predominance of distant vs. loco-regional relapse in patients operated for primary NSCLC, several aspects of the relapse pattern still have not been fully elucidated. Data about lung cancer relapse are usually added to long term survival data, mainly without details other than about the form of relapse [1,2]. There are few reports specifically addressing the pattern of relapse including exact onset of relapse, the way of detecting relapse (symptom based/controls) and treatment, taking account of tumour and patient related characteristics [3].

We set out to determine if intensified follow up of these patients could influence the outcome of treatment through earlier detection of relapse and initiation of treatment. Our hypothesis was that the reason for treatment failure in many operated patients, independently of the way of preoperative mediastinal assessment, could be the existence of clinically occult micrometastases at the time of operation, leading to early, unrecognized cancer relapse, usually with delayed, if with any specific treatment.

The aim of the study was to assess whether the intensified follow up of the operated patients contributes to the earlier treatment of relapse or indicates the way of improving the preoperative patient selection.

### Patients and methods

Prospective, controlled study that included 88 patients with complete lung resection for NSCLC in the period December 2002 - March 2004.

The mean age of patients was 55 years, ranging 42-77 years, M:F 6.3:1.

Stage IIIA existed in 35(39.8%) patients, whilst stages IB, IIA and IIB existed in 10.2%, 4.5% and 45.5% patients respectively.

In the present study, the 1997 revision of TNM system was used in order to determine the disease stage based on the operative specimens of the lung tissue and harvested lymph nodes.

#### Inclusion criteria

Stage I-IIIA; complete resection; systematic lymphadenectomy with at least 6 different lymph node groups examined; no neoadjuvant therapy; exact data about tumour histology, tumour diameter, grade of tumour differentiation, visceral pleural involvement, vascular and lymphatic invasion; regular monthly contacts with patients and written report about the patient's status; exact date of the relapse suspicion and confirmation; exact data about the site of relapse; evidence of pathologic confirmation of relapse; precise evidence about treatment of the relapse - date the treatment began and ended, form of the treatment; outcome of the treatment (alive and disease free, alive with disease, dead); date of death; cause of death.

#### Preoperative work up

Standard clinical and laboratory investigations, bronchoscopy, high-resolution CT of the thorax and upper abdomen, respiratory function tests, blood gasses in the arterial blood.

Mediastinoscopy was not routinely performed in the analysed period.

In patients with moderate to severe COPD), combined bronchodilator therapy, with or without antibiotics was applied. Patients with FEV_1_and 100 FEV_1_/VC greater than 60% at control spirometry. were referred directly to surgery. Patients with FEV_1_and 100 FEV_1_/VC lower than 60% at control spirometry, were subjected to perfusion scintigraphy of the lungs, in order to calculate the predicted postoperative FEV_1_(ppoFEV_1_). They were referred to surgery if their ppoFEV_1 _was greater than 30% predicted.

#### Follow up and data analysis

Follow up period: December 2002-December 2008.

In the analyzed group, an intensified follow+up was applied. The term "intensified follow up" relates to regular monthly phone contacts with patients and/or their families in order to get reliable information about the patient's general condition and eventual new complaints that were not present on discharge. Independently of this follow up, all the included patents were regularly controlled in the outpatient clinic at one month intervals during the first 3 months, than at 3 months intervals till the end of the first postoperative year. During the second and third postoperative year, intensified follow up was combined with regular outpatient controls at 4 months. Further outpatient controls were shaduled at 6 months intervals, combined with intensified follow up as described.

Data were collected from the original patients' hospital and outpatient dossiers and by contacting the patients or their relatives or physicians by phone. The obtained demographic and clinical data, including age, gender, pulmonary function, comorbidity, quality of life after the operation, as well as perioperative data, consisting of surgical procedure, pathologic stage, and operative morbidity and mortality, were entered into the database.

The overall and disease free survival were calculated, as well as local relapse free and distant metastases free survival. Disease free survival corresponds to the length of time after the operation during which a patient survives with no signs of disease.

Locoregional relapse-free interval represents the time interval (in months) between the operation and diagnosis of the locoregional relapse. As for this type of relapse, symptoms may not be reliable in terms of the existence of relapse. the moment of the relapse diagnosis by imaging and/or biopsy represented the moment of the relapse onset.

Distamt relapse-free survival refers to the time interval (in months) between the operation and detection of distant metastases. In patients with subsequently confirmed brain or bome metastases, the appearance of first specific symptoms was accepted as the time of the relapse onset.

Cancer relapse inside the first 12 postoperative months was particularly analysed.

Univariate and multivariate analysis of factors influencing the overall survival and the relapse occurrence included: interval between the onset of symptoms and operation, radiographic aspect, bronchoscopic aspect, tumour diameter, T and N stage, visceral pleural involvement, extent of resection and adjuvant treatment.

#### Statistics

T-test for independent samples was used to assess the influence of the Tu diameter to the length of the interval operation-relapse and to the pattern of relapse occurrence (inside the first postoperative year or later).

Chi-square test.was used to assess the influence of the percentage of N2 lesions to the length of the interval operation-relapse and to the occurrence of relapse inside the first postoperative year. year. Also this test was used to assess the distribution of N2 lesions depending of the type of T2 descriptor. Survival was estimated by the Kaplan Meier method.

Multivariate analysis of prognostic factors was performed via Cox proportional hazard regression with backward elimination until all remaining model parameters were significant at the 0.05 level.

## Results

### Structure of the analysed group

The mean age of patients was 55 years, ranging 42-77 years, M:F 6.3:1.

Stage IIIA existed in 35(39.8%) patients, whilst stages IB, IIA and IIB existed in 9(10.2%), 4(4.5%) and 40(45.5%) patients respectively.

There were 38 right sided and 50 left sided tumours. Thirty five patients underwent lobectomy and 53 underwent pneumonectomy.

In 67 (76%) patients squamous cell carcinoma existed. There were 18 patients with adenocarcinoma, one with bronchioloalveolar carcinoma and two with adenosquamous carcinoma. Postoperatively, 23(26.1%) patients underwent adjuvant therapy (21 irradiation and 2 chemotherapy).

### T stage; N stage

Of 70 patients with T2 tumors, tumour diameter (>3 cm) was the only T descriptor in 41(58.6%) patients, visceral pleural involvement was the only T descriptor in 2 patients, whilst in the remaining 27(38.6%) patients, both tumour diameter >3 cm and visceral pleural involvement existed (table 1). Five different T3 descriptors were almost equally distributed.

**Table 1:** T-descriptors

T2 tumours		
	**n**	**%**

Rtu > 3 cm	41	58.6

visceral pleura	2	2.8

Rtu> 3 cm + visceral pl.	27	38.6

total	70	100

**T3 tumours**	n	%

chest wall	4	28.6

parietal pleura	3	21.5

mediastinal pleura	2	14.2

pericardium	3	21.5

< 2 cm from carina	2	14.2

total	14	100

Mediastinal lymph node metastases existed in 12(29.35) patients with tumour diameter as the only T2 descriptor and in 9(33.3%) patients with visceral pleural involvement (P > 0.8136).

**Figure 1:**
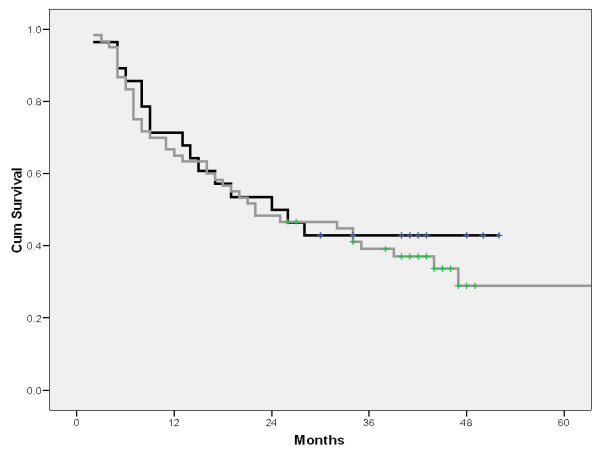
**Survival depending n visceral pleural involvement (gray line: visceral pleura intact, black line: visceral pleura invaded)**.

Although the survival of patients with T2 tumours and visceral pleural involvement was inferior vs. patients with intact visceral pleura, this survival difference was not statistically significant-median survival 34 ± 7 months vs. 26 ± 7 months (figure 1).

The percent of positivity of the examined mediastinal lymph nodes varied between 2.8% for pulmonary ligament nodes and 17.8% for the upper paratracheal nodes. Metastases in hilar, interlobar and lobar lymph nodes were confirmed in 29.5, 22.4 and 81.8% of examined lymph nodes.

### Pattern of relapse; disease free survival

The type of relapse according to stage is presented on table 2. During the follow up period, postoperative lung cancer relapse occurred in 50(56.8%) patients. In 44 patients symptoms existed, whilst in 6 asymptomatic patients relapse was detected at regular controls.

**Table 2:** Type of postoperative cancer relapse

*LC relapse*	n	%
with symptoms	44/50	88

without symptoms	6/50	12

locoregional	13/50	26

distant	35/50	70

locoreg.+ distant	2/50	4

relapse in IB	2/9	22.2

relapse in IIB	21/40	52.5

relapse in IIIA	27/35	77.1

relapse in pN0	3/10	30

relapse in pN1	30/54	55.6

relapse in pN2	17/24	70.8

Locoregional, distant and both types of relapse occurred in 26%, 70% and 4% patients respectively. Postoperative cancer relapse occurred in 27/35(77.1%) pts. in the stage IIIA and in 21/40(52.5) pts in the stage IIB. In none of four pts. in the stage IIA cancer relapse occurred, unlike 22.2% pts. with relapse in the stage IB.

In patients with N0, N1 and N2 lesions, cancer relapse occurred in 30, 55.6 and 70.8% patients respectively.

Cancer relapse occurred in 37/67(55.2%) patients with sqiamous cell carcinoma and in 13/21(62%) patients with other cell types.

In patients with relapse, well differentiated, moderately and poorly differentiated tumours existed in 10(20%), 25(50%) and 15(30%) patients respectively. In patients without relapse, the same categories of the grade of tumour differentiation existed in 6(15.9%), 24(63.1%) and 8(21%) patients respectively.

The overall two, three and 5 year survival was 48.8, 40.2 and 33% respectively.

**Figure 2:**
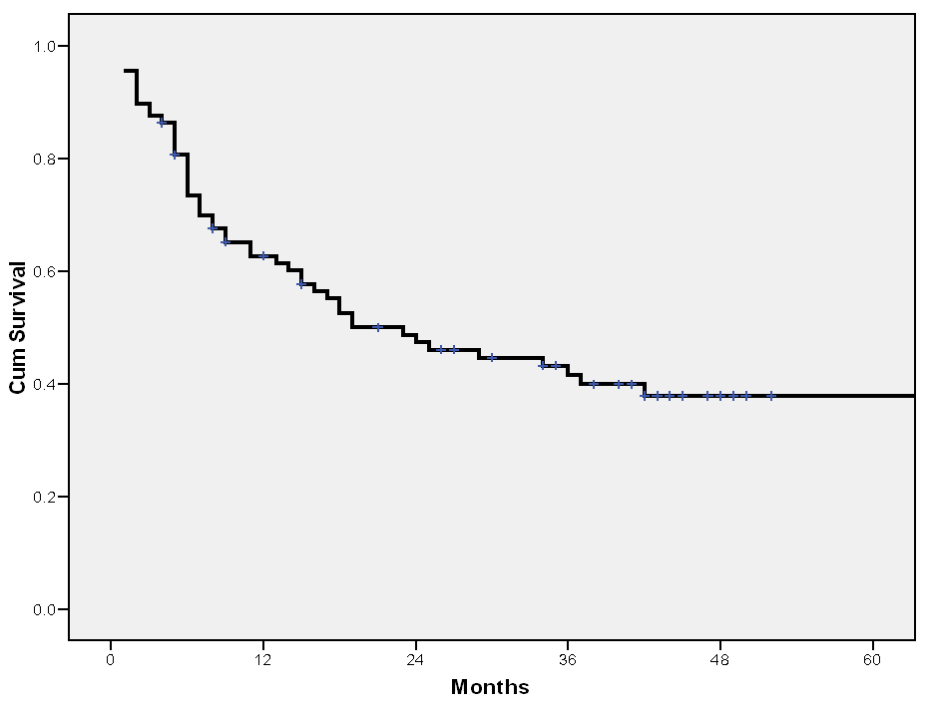
**Disease-free survival**.

The disease free survival is presented on the figure 2. One year after the operation, 62.7% patients were alive and disease free. Two years after the operation 47.3% patients were disease free, whilst the percentage of patients without relapse decreased to 41.5 and 37.7% three and four years after the operation.

The overall disease free interval, local relapse-free and distant relapse-free intervals are presented on the table 3. The mean disease-free interval in the analysed group was 34.4 ± 3.2 months (median 19, 95%CI: 6.62-31.38). The mean local relapse-free and distant relapse-free intervals were 55 ± 3.3 and 41.6 ± 3.5 months respectively.

**Table 3:** Disease-free intervals

disease free interval (months)						
	**mean**	**SE**	**95% CI**	**median**	**SE**	**95% CI**

**overall**	34.4	3.2	28.0-40.77	19.0	6.31	6.62-31.38

**locrelapse-free**	55	3.3	48.49-61.52	*	*	*

**dist. relapse-free**	41.6	3.5	34.83-48.42	*	*	*

*: median not presented (> 50% alive after 70 months postoperatively)

SE: standard error; CI: confidence interval

loc.relapse-free: interval without locoregional relapse

dist. relapse-free: interval without distant relapse

**Figure 3:**
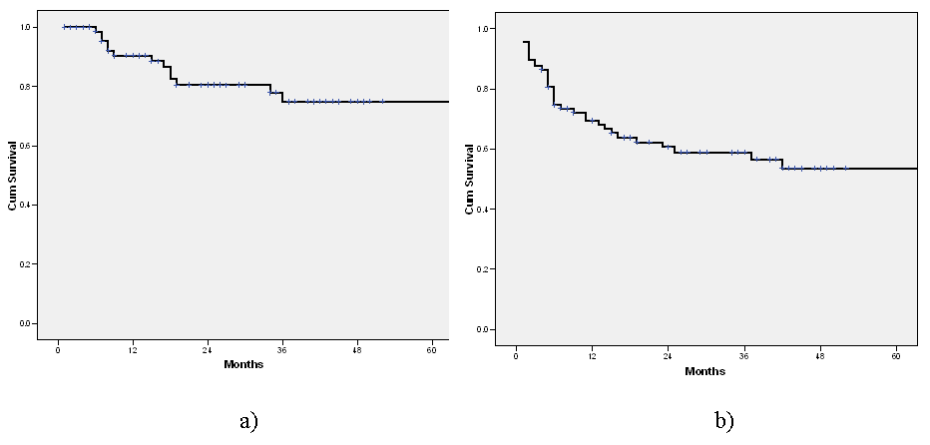
**Locoregional (a) and distant (b) relapse-free survival**.

One year after the operation, 90.4% patients were without locoregional relapse. Two years after the operation 78.3% patients were locoregional relapse free, whilst this percentage decreased to 70.7% four years after the operation (figure 3a). The percentage of distant relapse free patients decreased from 69.4 ± 5% one year after the operation, to 50.5 ± 6.2% four years after the operation (figure 3b).

### Relapse inside the first postoperative year

Among 30 pts. with the relapse onset inside the first 12 month after the lung resection, the disease free interval <3 months, 3-6 months and >6 months occurred in 10(33.3%), 12(40%) and 8(26.7%) pts. respectively. In 20(66.6%) pts. in this group, either T3 tumours or N2 lesions existed (table 4). Although the tumour diameter in patients with relapse inside the first postoperative year was 79 ± 32 mm vs. 63 ± 19.8 mm in patients with later relapse occurrence, this difference was not statistically significant. Mediastinal lymph node metastases existed in 40.6% patients with relapse inside the first year vs. 22.2% patients with N2 lesions and relapse after the first year (P: 0.395).

**Table 4:** Relapse during the first postoperative year

disease-free interval (months)	< 3		3-6		>6	
	
	n	%	n	%	n	%
	
	10	33.3	12	40	8	26.7
	**interval operation-relapse**					

	**< 1 year**			**> 1 year**		

**Tu diameter (mm)**	79 ± 32			63 ± 19.8		

**N2 (%)**	40.6			22.2		

***P value***	**Rtu: > 0.065; N2: 0.39**					

### Distant metastases

Among 37 patients with metastases, one single and more than one distant sites existed in 70.27% and in 29.73% patients respectively. The most frequent distant site was brain (51.4% pts), followed by bone (18.9% pts.), liver and contralateral lung (16.2% pts. each). Metastases in distant lymph nodes, adrenals and other sites were registered in five, three and two patients respectively.

Although the median disease free interval of 3 months (95% CI: 0.39-5.61) in patients with brain metastases was shorter than the same interval of 6 months in patients with metastases in other sites, this difference is not statistically significant (P: 0.0735).

### Extent of resection; relapse treatment

**Figure 4:**
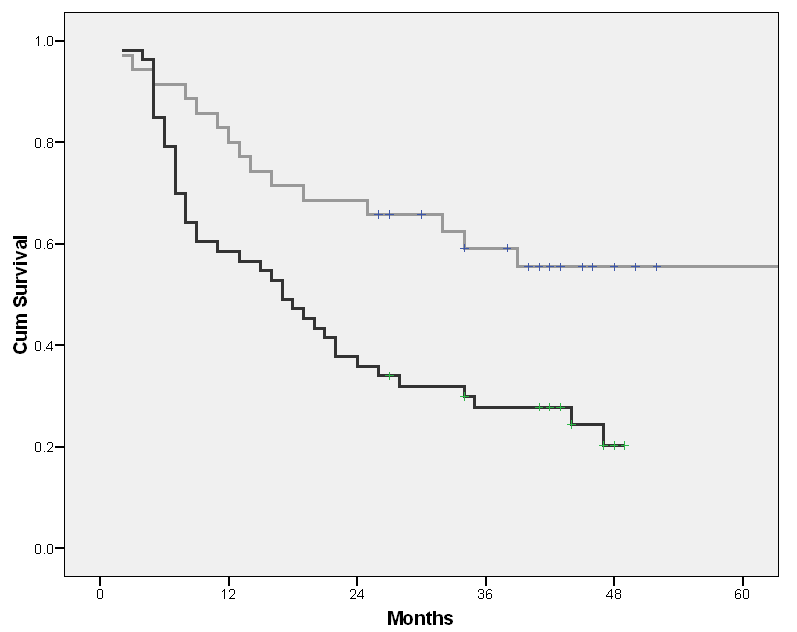
**Survival depending on the extent of resection (gray line: lobectomy, black line: pneumonectomy)**.

The median disease free interval after lobectomy was 36 months vs. 16 onths after pneumonectomy (P: 0.0925). Survival after lobectomy was significantly longer than after pneumonectomy (figure 4).

Specific oncological treatment of relapse was possible in 27/50(54%) patients. Fourteen patients underwent radiation therapy only; in 9 patients operative treatment was performed, either as the only treatment modality (two patients) or in combination (5 OP+RT, 2 OP+CT+RT). Chemotherapy alone was given to two patients, whilst one patient underwent chemotherapy combined with radiation therapy.

### Univariate and multivariate analysis of prognostic factors

Univariate analysis of factors influencing survival and relapse occurrence are presented on the table 5. Radiographic aspect (tumour shadows vs. other categories), T stage (<T3 vs T3), N stage (N0/N1vs. N2) and extent of resection (lobectomy vs. pneumonectomy) were found as significant in terms of survival. Symptom duration longer than three months (vs. < 3 months) and tumour diameter <5 cm (vs. >5 cm) were associated with better survival, but without statistical significance.

**Table 5:** Univariate and multivariate analysis of factors influencing survival and relapse

		UNIVARIATE						MULTIVARIATE		
		**survival (months)**			**relapse free interval (months)**			**survival**		

		***median***	***95% CI***	***P***	***median***	***95% CI***	***P***	**SE**	**df**	**sig**.

**Rtg aspect**	Tumour other categories)	44 16	26.12-61.88 8.59-23.41	0.0289	42 15	32.16-51.84 7.15-22.85	0.0557	0.361	1	0.743

**T component**	< T3 T3	34 9.0	18.77-49.23 3.50-14.50	0.0007	34 8	17.87-50.13 5.92-10.08	0.0064	0.388	1	0.002

**N component**	<N2 N2	34 8.0	13.70-54.29 5.61-10.39	0.001	34 6.0	14.17-53.82 0.0-13.98	0.002	0.291	1	0.019

**Extent of resection**	lobectomy pneumonectomy	.* 17	.* 9.87-24.13	0.0925	36 16	23.57-48.13 7.89-24.11	0.0925	0.477	1	0.298

**Duration of symptoms** **(months)**	**< 3** > 3	18 32	10.67-25.33 30.62-53.46	0.0623	17 37	6.83-27.17 21.28-52.42	0.1645	0.344	1	0.180

**Bronchoscopic aspect**	normal pathologic	26 22	0.00-54.51 12.42-31.58	0.7294	29 19	1.91-56.09 8.88-29.12	0.6950	0.308	1	0.579

**Visceral pleura**	invaded intact	24 22	9.74-38.26 5.88-38.12	0.5788	36 19	25.42-46.58 8.89-29.11	0.5094	0.362	1	0.598

*: median not presented (> 50% alive after 70 months postoperatively)

Related to the relapse occurrence, although radiographic aspect and extent of resection followed the same trend as in the survival analysis, only T stage and N stage were found as significant in the same sense as for survival.

On multivariate, only T and N stage were found as significant in terms of survival.

## Discussion

The main point of the present study is reliability of data owing to regular short interval contacts with patients. So, the obtained relapse pattern can be considered highly reliable and pure, i.e. uninfluenced by nonsurgical treatment owing to the absence of patients with neoadjuvant treatment. This could justify such a study design and expected practical benefit. Moreover, although several factors have been shown to affect survival, few studies have demonstrated any correlation between these factors and tumor recurrence. In fact, most studies focused on survival as end point [4-6].

The type of lymphadenectomy in the present study was complete removal of all palpable and visible lymph nodes. Most of our patients had seven or more groups of lymph node stations harvested. It was clearly demonstrated that, after less than 4, 4-6, 7-9 nodes harvested, the corresponding 5-year disease-free survival rates were 43.4%, 67.3% and 76.3% respectively [7]. It was also recently shown that sampling adequately recognized N2 disease and multilevel N2 lesions in only 52% and 40% patients respectively [8]. A certain recently expressed concerns related to the influence of tumor side (the aforementioned advantages of dissection could not always be confirmed in presence of left sided tumors) [9], did not influence our current policy of lymphadenectomy.

The reason for particularly analyzing T2 descriptors lies in the results of some recent studies which found visceral pleural involvement as significant prognostic factor [10]. Despite the nearly equal percent of mediastinal lymph node metastases in our patients with and without visceral pleural involvement, a clear trend of survival worsening was found if the visceral pleura was invaded. However, due to the absence of statistical significance (median survival 34 ± 7 vs. 26 ± 7 months), our results are in line with studies demonstrating that, even in the stage I, the prognostic significance of non-size based T2 descriptors depends on tumor size. By the other hand, it was clearly shown that, by not taking account of different T2 descriptors, as many as 21.1% of the stage IB patients may be unnecessarily upstaged from stage IA to stage IB as their survival was not different from that of stage IA patients [11].

The causes of a quite high relapse rate (56.8%) in the present study during the follow up period can be discussed from several aspects. First, the predominance of distant vs. locoregional type is an expected finding, usually explained by the variability in the pattern of lymphatic drainage and an incidence of skip metastases of 31-74% [12]. Moreover, microscopic metastases in the N1 and N2 nodes are often below the limits of detection by PET [13,14]. Second, in the majority of our patients with relapse (30/50), relapse occurred inside the first 12 months after the operation, in 22/30 less than 6 months after the operation. Such a finding clearly indicates the existence of distant metastases in these patients at the time of the operation, thus supporting the evidence of distant metastases at the moment of operation as one of major causes of understaging, even in the stage I [15]. Third, greater tumour diameter in our patients with relapse during the first 12 months vs. patients with later relapse (79 ± 32 vs.63 ± 19.8 mm) underlines the role of the tumour diameter, thus supporting observations that there is a three-fold increase in the risk of having pathologic stage II or stage III disease with every 1.0 cm increase in tumor size [16]. But, it is also true that the diameter after which the risk begins to increase has not still been defined. The association of the greater tumour diameter with higher percentage of N2 lesions compared with smaller tumours (40.6 vs. 22.2%) in our study, represents the reflexion of already described doubling of risk for occult N2 lesions with the increase of the tumour size from <1 cm to over 2 cm [17].

Despite the evidently higher proportion of local relapse-free vs. distant relapse-free patients after the first (90.4 vs. 69.4%) and second postoperative year (78.3 vs. 50.5%), the mean local relapse free and distant relapse free intervals were not significantly different. Nevertheless, this evident difference during the first postoperative year clearly reflects the relatively high percent of patients with early relapse. As most of them had asymptomatic brain and bone metastases, with around a third with more than one metastatic sites, the way of extrathoracic assessment of the disease can be put into question. It is clear that it should be intensified, but based on the obtained results, (mostly because of limited patient number), it is not possible to conclude with certainty in which subset of patients it should be done. It is now well known that the proportion of patients with unexpected extrathoracic metastases varies between 5-29% and that, even PET scan, (although better than CT and bone scintigraphy in detecting liver and bone metastases) is not a good technique in the search for brain metastases [18,19]. Moreover, it was also shown that even the combination of CT, bone scintigraphy, abdominal ultrasonography and PET scan still misses micrometastases in about 20% of patients [20].

In brief, our expectation that this intensified follow up will help to discover relapse in more asymptomatic patients than without it was not confirmed, because in 88% of patients the relapse was discovered because of symptoms and only in 12% at controls. Furthermore, a specific oncological treatment (that could be the end point of this intensified follow up) was performed in only a half of the operated patients. Similarly, the overall survival of patients with relapse discovered by this form of follow up was not different from usually reported rates in the literature. It means that, in this subset of patients, survival is not subject to the influence neither of lead time bias, nor of length time bias, that could cause the false impression of prolonged survival associated with more frequent patients' controls, that sometimes occurs in some screening protocols.

When discussing unexpected relapse in our patients, it should be mentioned that micro metastases to small nodes without mediastinal nodes enlargement is reported to occur in 8-60% pts. with mediastinal metastases [21,22]. In the analysed group, relapse occurred in patients with N0 and N1 lesions in 30 and 55.6% patients respectively. Such a finding supports the significance of analysis of extranodal extension, because it was demonstrated that the 5-year survival rate of stage IIIA patients without extranodal extension could be significantly better than that of stage II patients with extranodal extension - 30.4 vs. 16.8% in some series [23]. Beside extranodal extension, a possible cause of at least a part of relapse in presence of N1 can be due to metastase in nonprimary lobe nodes. Like in our study, metastases in these nodes are not frequently analysed and are reported to occur in up to 30% patients with lobar lymph nodes metastases [24]. Finally, as the higher proportion of relapse among patients with adenocarcinoma was an expected finding, the grade of tumour differentiation was expected to explain at least a part of the relapse pattern in this study. But, the higher proportion of poorly differentiated tumours in patients with vs. patients without relapse (30% vs. 21%), was counterweighed by a smaller proportion of well differentiated tumours in patients without vs. patients with relapse (15.9% vs. 20%). Furthermore, as in both groups moderately differentiated tumours were dominant, the influence of this factor (probably because of the limited number of patients) requires further analysis.

Related to the extent of resection, we share the opinion of the authors stating that the analysis of the recurrence rate is likely to be more reasonable than the survival analysis, probably because of the more advanced stage and higher mortality in patients undergoing pneumonectomy. In the present study, despite the clearly longer mean disease free interval in the lobectomy group (41.1 ± 5 vs. 23.38 ± 2.8 months), due to the absence of statistical significance, our results are brought in line with those studies which did not confirm significantly different reccurence rate between these two groups [25].

Concerning prognostic factors at univariate analysis, the significant influence of the radiographic aspect (tumour shadows vs. other) was not accompanied by similar role of the bronchoscopic aspect as could be expected and as was demonstrated in some studies with lower local recurrence and higher survival rates associated with positive preoperative bronchoscopic findings. Moreover, although one could expect that aspect other than tumour shadows (atelectasis, hilar masses) could be associated with better survival because of probable existence of more symptoms (secreion retention, hemoptysis), the situation was the opposite in our study. The probable cause is the greater proportion of tumours with diameter > 8 cm in our group, than in most of the reported series. Nevertheless, this factor was not revealed as significant related to recurrence, just like the influence of the extent of resection, that was discussed above.

The fact that only T and N factors were found as significant at multivariate, is not unexpected, once again underlining the role of proper patient selection.

One potential limitation of this study is our intentional omission of the control group. The reason for that is the fact that, even in case controled study, the reliability of data related to many aspects of relapse in any control group, if obtained retrospectively, could cause many biases if compared with the presented relapse pattern obtained in a prospective manner.

As a conclusion, this study showed that the intensified follow up did not increase either the proportion of patients detected with asymptomatic relapse or the number of patients with specific oncological treatment of relapse.

## Competing interests

The authors declare that they have no competing interests.

## Authors' contributions

DS conceived of the study, and participated in its design and coordination; he also operated the majority of patients included in the study. DM participated in the study design; he also operated a part of patients included in the study. GR was in charge for the intensified follow up of operated patients and for the coordination of the study. JS participated in the study design, and was responsible for patohistological diagnosis in all patients included in the study. MG performed the statistical analysis. ME participated in the study design; directly responsable for the immediate and early postoperative course in all patients included in the study. All authors read and approved the final manuscript.
